# Utility of non-HDL-C and apoB targets in the context of new more aggressive lipid guidelines

**DOI:** 10.1016/j.ajpc.2021.100203

**Published:** 2021-05-29

**Authors:** Renato Quispe, Adam J. Brownstein, Vasanth Sathiyakumar, Jihwan Park, Blair Chang, Aparna Sajja, Eliseo Guallar, Mariana Lazo, Steven R. Jones, Seth S. Martin

**Affiliations:** a Ciccarone Center for the Prevention of Cardiovascular Disease, Johns Hopkins Hospital, 600 N. Wolfe St, Carnegie 591, Baltimore 21287 MD, United States; b Division of Cardiology, Department of Medicine, Johns Hopkins University School of Medicine, Baltimore MD, United States; c Department of Medicine, Johns Hopkins Hospital, Baltimore MD, United States; d Johns Hopkins Bloomberg School of Public Heath, Baltimore MD, United States; e Johns Hopkins University, Baltimore MD, United States; f Welch Center for Prevention, Epidemiology, and Clinical Research, Baltimore MD, United States

**Keywords:** Low-density lipoprotein cholesterol, Non-high-density lipoprotein cholesterol, Apolipoprotein B

## Abstract

**Objective:**

Major guidelines recommend the use of secondary targets, such as non-HDL-C and apoB, to further reduce cardiovascular risk. We aimed to evaluate the proportion at which newer, more aggressive secondary lipid targets are exceeded in patients with LDL-C < 70 mg/dL estimated by Friedewald (LDLf-C) and Martin/Hopkins equations (LDLm-C).

**Methods:**

We analyzed patients from the Very Large Database of Lipids with fasting lipids and estimated LDL-C <70 mg/dL by the Friedewald equation and Martin/Hopkins algorithm. Patients were categorized into three groups: LDL-C <40, 40–54 and 55–69 mg/dL. We calculated the proportion of patients with non-HDL-C and apoB above high-risk targets (non-HDL-C ≥ 100 and apoB ≥ 80mg/dL) for those with LDL-C 55-69 mg/dL and very high-risk targets (non-HDL-C ≥ 85 and apoB ≥ 65mg/dL) for those with LDL-C < 40 mg/dL and 40-54 mg/dL.

**Results:**

In patients with LDLf-C < 40 mg/dL, ~8 and ~4% did not meet high-risk secondary targets and ~21 and 25% did not meet very high-risk secondary targets for non-HDL-C and apoB, respectively. However, in patients with LDLm-C < 40 mg/dL <1% did not meet high-risk targets, while only 3% did not meet the very-high risk secondary target for apoB and none exceeded the very-high risk secondary target for non-HDL-C. Among individuals with LDL-C< 40 mg/dL, there were increasing proportions of individuals not meeting the very high-risk secondary apoB target at greater triglyceride levels, reaching up to ~19% using LDLm-C compared to ~60% using LDLf-C when triglyceride levels were 200–399 mg/dL. There were higher proportions of individuals not meeting high and very-high risk targets as triglyceride levels increased among those with LDL-C 40–54 and 55–69 mg/dL.

**Conclusion:**

In a large, US cross-sectional sample of individuals with LDL-C < 70 mg/dL, secondary non-HDL-C and apoB targets overall provide modest utility. However, attainment of very high-risk cutpoints for non-HDL-C and apoB is not achieved in a significant fraction of patients with triglycerides 200–399 mg/dL, even when using a more accurate calculation of LDL-C.

## Introduction

Recent data have supported more aggressive lowering of low-density lipoprotein-cholesterol (LDL-C) for management of atherosclerotic cardiovascular disease (ASCVD) [1,2]. The European Society of Cardiology/European Atherosclerosis Society (ESC/EAS) guidelines recommend targeting an LDL-C goal of <70 mg/dL in high risk patients, <55 mg/dL in very-high risk patients, and <40 mg/dL in very-high risk patients with a second vascular event within 2 years, while the American Heart Association/American College of Cardiology guidelines use an LDL-C threshold of 70 mg/dL to consider starting non-statins [1,2]. Given that apolipoprotein B (apoB) and non-high-density lipoprotein-cholesterol (non-HDL-C) may be complementary to LDL-C [1], clinicians may turn to these to further decrease ASCVD risk once LDL-C is optimized. The extent to which non-HDL-C and apoB remain uncontrolled will determine the extent to which lipid-lowering therapy may be further intensified and risk further lowered.

We have previously demonstrated that after using the Martin/Hopkins algorithm, which is more accurate than the Friedewald method [3], secondary targets for non-HDL-C (<100 mg/dL) and apoB (<80 mg/dL) were not met in only ~1–2% of the population [4]. The ESC/EAS guidelines proposed updated secondary targets for very high- and high-risk patients: non-HDL-C < 85 and <100 mg/dL, and apoB <65 and < 80 mg/dL, respectively [2]. Since we have entered an era of more intensive lipid-lowering therapy, we sought to extend our prior work by performing an analysis comparing attainment of these new lipid targets.

## Methods

### Study population

We analyzed patients from the Very Large Database of Lipids with complete fasting lipids (including a standard lipid panel and apoB) and estimated LDL-C < 70 mg/dL by the Friedewald equation (LDLf-C; *n* = 11,220; 43.4% female; median age 66 years, IQR 54-74) and Martin/Hopkins algorithm (LDLm-C; *n* = 9,189; 44.1% female, median age 66 years, IQR 54-75). As detailed previously, the Very Large Database of Lipids consists of deidentified data from U.S. patients referred for lipid testing primarily from outpatient primary care clinics [4]. Our study was declared exempt by the Johns Hopkins Institutional Review Board because we used only deidentified data routinely collected during clinical lipid measurements.

### Lipid measurements

Vertical auto profile (VAP), a rapid ultracentrifugation technique that separates lipoproteins in <1 h, was used to measure the cholesterol content in lipoprotein fractions. Triglycerides (TG) were measured with the Abbott. ARCHITECT C-8000 system, and apoB was measured with an Abbott ARCHITECT analyzer and reagent in accordance with World Health Organization standards.

### Statistical analysis

Friedewald-estimated LDL-C was calculated as total cholesterol minus HDL-C minus TG/5 in mg/dL. The Martin/Hopkins equation was calculated as total cholesterol minus HDL-C minus TG/adjustable factor in mg/dL, whereby 1 of 174 patient-specific ratios for very low-density lipoprotein cholesterol (VLDL-C) estimation were set by non-HDL-C and TG categories [3]. We performed a separate analysis for LDLf-C and LDLm-C. We stratified patients into three LDL-C categories: < 40, 40–54, and 55–69 mg/dL to reflect the targets recommended in the ESC/EAS guidelines. Within each LDL-C category, we calculated the proportion of patients who had non-HDL-C and apoB levels above guideline cutpoints for high-risk patients (≥100 mg/dL and ≥80 mg/dL, respectively). Among those with LDL-C <55 mg/dL, we performed the same analysis using the very-high risk cutpoints for non-HDL-C and apoB proposed in the ESC/EAS guidelines (≥85 mg/dL and 65 mg/dL, respectively). We then stratified patients within each LDL-C category by TG levels (TG < 100 mg/dL, 100–199 mg/dL, and 200–399 mg/dL) and performed the same analysis as above for high and very-high risk cutpoints. Table 1.

**Table 1:** Absolute number and percentage of patients stratified by either Friedewald or Martin/Hopkins LDL-C and triglyceride levels who did not meet the ESC/EAS high risk or very-high risk secondary targets for non-HDL-C (<100 and 85 mg/dl, respectively) or apoB (<80 and 65 mg/dL, respectively).

		**Absolute No. (%) of patients with TG <100** **mg/dL**	**Absolute No. (%) of patients with TG 100–199** **mg/dL**	**Absolute No. (%) of patients with TG 200–399** **mg/dL**
**Martin/Hopkins method**				
**LDLm-C** **<** **40** **mg/dL**	No. of patients	277	113	26
	non-HDL-C ≥ 85	0 (0%)	0 (0%)	0 (0%)
	ApoB ≥ 65	2 (0.72%)	4 (3.54%)	5 (19.23%)
**LDLm-C 40–54** **mg/dL**	No. of patients	1307	684	124
	non-HDL-C ≥ 85	0 (0%)	0 (0%)	20 (16.13%)
	ApoB ≥ 65	5 (0.38%)	59 (8.63%)	51 (41.13%)
**LDLm-C 55–69** **mg/dL**	No. of patients	3947	2223	488
	non-HDL-C ≥ 100	0 (0%)	0 (0%)	182 (37.30%)
	ApoB ≥ 80	3 (0.08%)	18 (0.81%)	71 (14.55%)
**Friedewald method**				
**LDLf-C** **<** **40** **mg/dL**	No. of patients	233	236	299
	non-HDL-C ≥ 85	0 (0%)	0 (0%)	163 (54.52%)
	ApoB ≥ 65	2 (0.86%)	16 (6.78%)	179 (59.87%)
**LDLf-C 40–54** **mg/dL**	No. of patients	1208	1145	497
	non-HDL-C ≥ 85	0 (0%)	170 (14.85%)	490 (98.59%)
	ApoB ≥ 65	5 (0.41%)	242 (21.14%)	433 (87.12%)
**LDLf-C 55–69** **mg/dL**	No. of patients	3678	2931	993
	nonHDL ≥ 100	0 (0%)	344 (11.74%)	957 (96.37%)
	apoB ≥ 80	4 (0.11%)	111 (3.79%)	544 (54.78%)

## Results

**Fig. 1:**
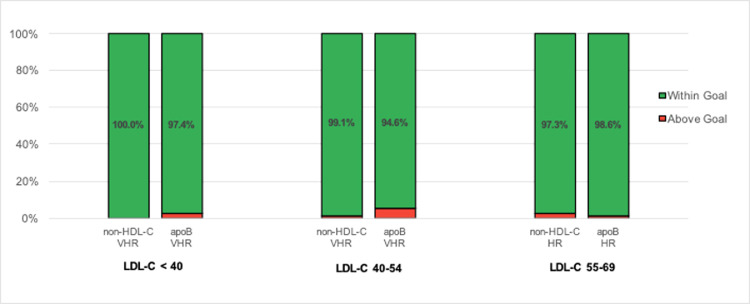
Percentage of patients with non-HDL-C and apoB within and above goal stratified by LDL-C values calculated by the Martin/Hopkins equation. *VHR: very-high risk; HR: high risk.*

**Fig. 2:**
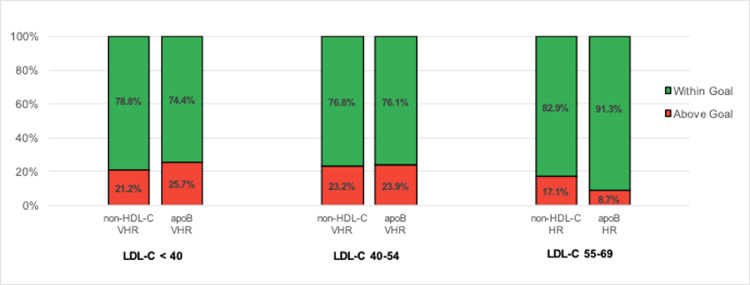
Percentage of patients with non-HDL-C and apoB within and above goal stratified by LDL-C values calculated by the Friedewald Equation. *VHR: very-high risk; HR: high risk.*

In patients with LDLf-C <40 mg/dL (*n* = 768), ~8% and ~4% did not meet secondary targets of non-HDL-C < 100 mg/dL and apoB < 80 mg/dL, while <1% of those with LDLm-C < 40 mg/dL (*n* = 416) did not meet these targets. Additionally, no patients with LDLm-C < 40 mg/dL exceeded the very-high risk secondary target for non-HDL-C of 85 mg/dL, while ~3% did so for the very high-risk secondary target for apoB of 65 mg/dL. Among those with LDLm-C < 40 mg/dL, we found increasing proportions of patients not meeting the very high-risk secondary apoB target at greater TG levels, up to ~19% using LDLm-C compared with ~60% using LDLf-C at TG 200–399 mg/dL. Fig. 1 depicts the percentage of patients with non-HDL-C and apoB above goal stratified by the Martin/Hopkins equation, while Fig. 2 depicts the same for the Friedewald equation. Table 1 lists the absolute number and percentage of patients stratified by LDL-C level and TG triglyceride level not meeting secondary targets. Supplementary Table 1 shows the median non-HDL-C and apoB values among the total population and among patients who did not meet both high-risk and very high-risk secondary targets.

In patients with LDLf-C 40–54 mg/dL (*n* = 2,850), ~10% and ~6% did not meet the high-risk secondary targets for non-HDL-C and apoB, respectively, while <1% of those with LDLm-C 40–54 mg/dL (*n* = 2,115) did not meet these targets. Additionally, 1% and ~5% with LDLm-C 40–54 mg/dL did not meet the very-high risk secondary targets for non-HDL-C and apoB, respectively. In this LDL-C category, we observed similar increasing trends of proportions of patients not meeting the very high-risk secondary apoB target at greater TG levels, increasing to 87% for LDLf-C and 41% for LDLm-C at TG levels of 200–399 mg/dL. Supplementary Table 2 demonstrates that among patients with LDLm-C 40–54 mg/dL who did not meet very-high secondary targets, 80% were within 5 mg/dL above goal for non-HDL-C, while 63% were within 5 mg/dL above goal for apoB.

Finally, among those with LDLf-C 55–69 mg/dL (*n* = 7,602), ~17% and ~9% did not meet the high-risk targets for non-HDL-C and apoB, while ~3% and ~1% of those with LDLm-C 55–69 mg/dL (*n* = 6658) did not meet these targets, respectively. Less than 1% of patients with LDLm-C 55–69 mg/dL and TG < 200 mg/dL did not meet the secondary targets. However, ~37% and ~15% with TG 200-399 mg/dL did not meet the targets for non-HDL-C and apoB, respectively. The majority of patients not meeting secondary targets were within 5 mg/dL above goal (65% for non-HDL-C and 71% for apoB), as shown in Supplementary Table 2. The proportions of patients not meeting secondary targets significantly increased at greater TG levels for both LDLm-C (Supplemental Figure 1) and LDLf-C (Supplemental Figure 2).

## Discussion

Our findings demonstrate that when LDL-C is controlled to very low levels, non-HDL-C and apoB are also controlled in the vast majority of patients. Even among patients who exceed the secondary targets, the majority are within 5 mg/dL above goal. However, when new very-high risk cutoffs from the ESC/EAS guidelines are applied in patients with TG 200–399 mg/dL, non-HDL-C and apoB remain elevated in ~15-40% of patients even following more accurate calculation of LDL-C via the Martin/Hopkins method. Many patients treated with PCSK9 inhibitors attain LDL-C levels < 40 mg/dL (median was 30 mg/dL in FOURIER [5]), and in this group, we found that when using LDLm-C no patients exceeded the very-high risk non-HDL-C secondary target and only 3% overall exceeded the very-high risk apoB secondary target of 65 mg/dL. However, in the subgroup with TG 200–399 mg/dL, 1 in 5 patients had apoB ≥ 65 mg/dL. In such patients, even with tight LDL-C control, apoB may provide complementary information and could be a clinically actionable result used in shared decision making to consider additional intensification of therapy.

### Strengths and limitations

The sample size of the Very Large Database of Lipids dataset allows for thorough examination of granular data on a large scale among individuals with low and very low LDL-C levels. However, it is important to acknowledge some limitations in our study. The Very Large Database of Lipids does not include information on patient medications or clinical data other than age or sex. The lack of information on medication usage in the VLDL database may limit the generalizability of these results as the lipid profile of individuals on statin medications and ezetimibe with LDL-C values < 70 mg/dL may differ from those who are not on these medications.

## Conclusions

In summary, in a large, US cross-sectional sample, non-HDL-C and apoB overall provide modest utility in patients with very low LDL-C when using the Martin/Hopkins method. Utility increases substantially in the patient subgroup with TG 200–399 mg/dL, particularly for apoB when applying very-high risk ESC/EAS cutoffs.

## Funding

The Very Large Database of Lipids is supported by the David and June Trone Family Foundation.

## Authorship contribution statement

**Renato Quispe:** Conceptualization, Data analysis, Methodology, Writing of original draft, review and editing. **Adam J. Brownstein:** Conceptualization, Data analysis, Methodology, Writing of original draft, review and editing. **Vasanth Sathiyakumar:** Writing – review and editing. **Jihwan Park:** Data analysis, Writing – review and editing. **Blair Chang:** Writing – review and editing, **Aparna Sajja:** Writing – review and editing, **Eliseo Guallar:** Writing – review and editing, **Mariana Lazo:** Writing – review and editing, **Steven R. Jones**: Writing – review and editing, **Seth S. Martin:** Supervision, Conceptualization, Methodology, Writing of original draft, review and editing

## Declaration of Competing Interest

Drs. Martin and Jones are listed as coinventors on a pending patent filed by Johns Hopkins University for LDL‐C estimation using the method applied in this manuscript. Dr Jones has served as an advisor to Sanofi/Regeneron. Dr. Martin is supported by the American Heart Association (20SFRN35380046 and COVID19-811000), PCORI (ME-2019C1-15328), NIH (P01 HL108800), the David and June Trone Family Foundation, and the Pollin Digital Health Innovation Fund. He has served as a consultant in the past 24 months to AstraZeneca, Amgen, DalCor Pharmaceuticals, Esperion, Kaneka, Sanofi, and 89bio. He is a founder of and holds equity in Corrie Health, which intends to further develop the platform. This arrangement has been reviewed and approved by the Johns Hopkins University in accordance with its conflict of interest policies. The remaining authors have no disclosures to report.

## Appendix Supplementary materials

Image, application 1
