# Supplementary material for: Utility of non-HDL-C and apoB targets in the context of new more aggressive lipid guidelines
Source: Am J Prev Cardiol. 2021 May 29;7:100203. doi: 10.1016/j.ajpc.2021.100203 (PMC8387302; doi:10.1016/j.ajpc.2021.100203)
Supplement: Supplementary file 1 [file mmc1.docx]

**Supplemental Figure 1.** Percentage of patients with LDLm-C <70 mg/dL not meeting secondary targets across TG categories.


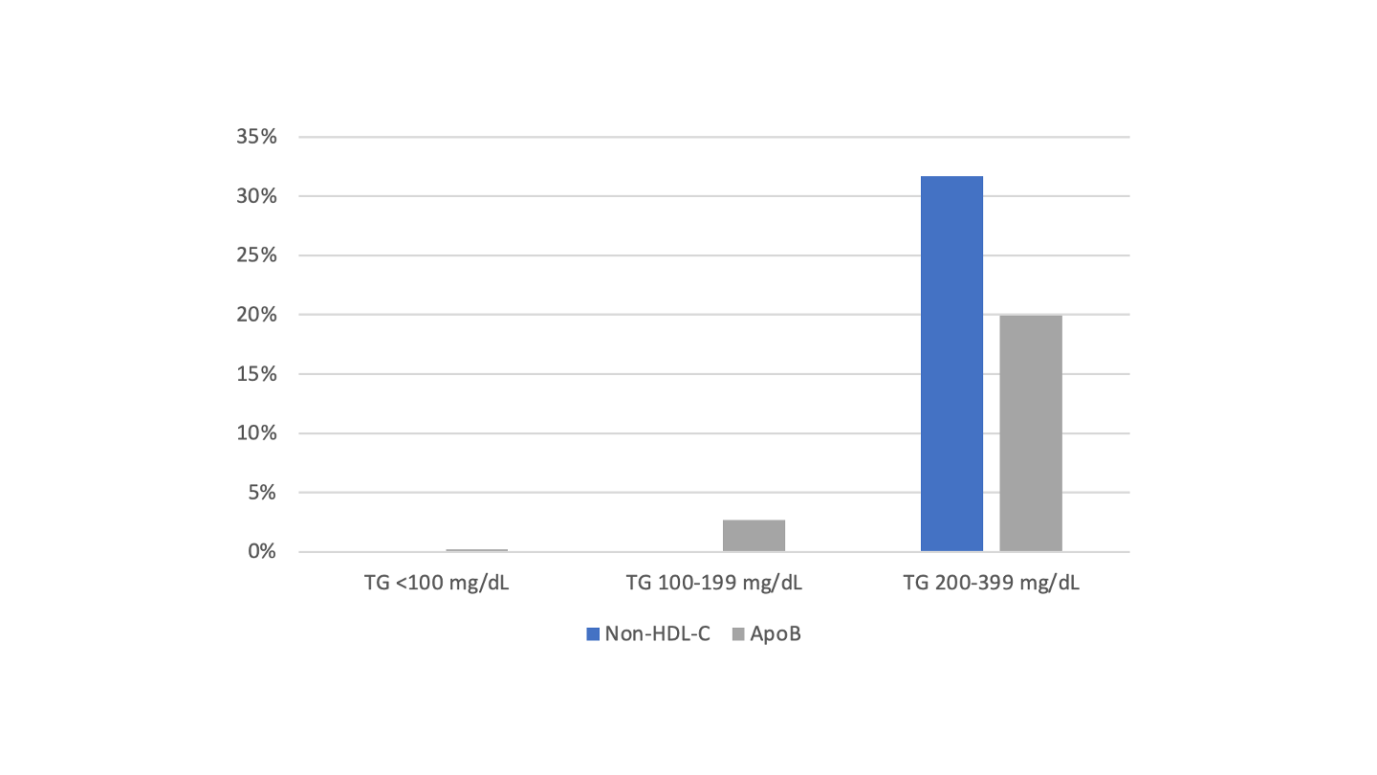


**Supplemental Figure 2.** Percentage of patients with LDLf-C <70 mg/dL not meeting secondary targets across TG categories.

**
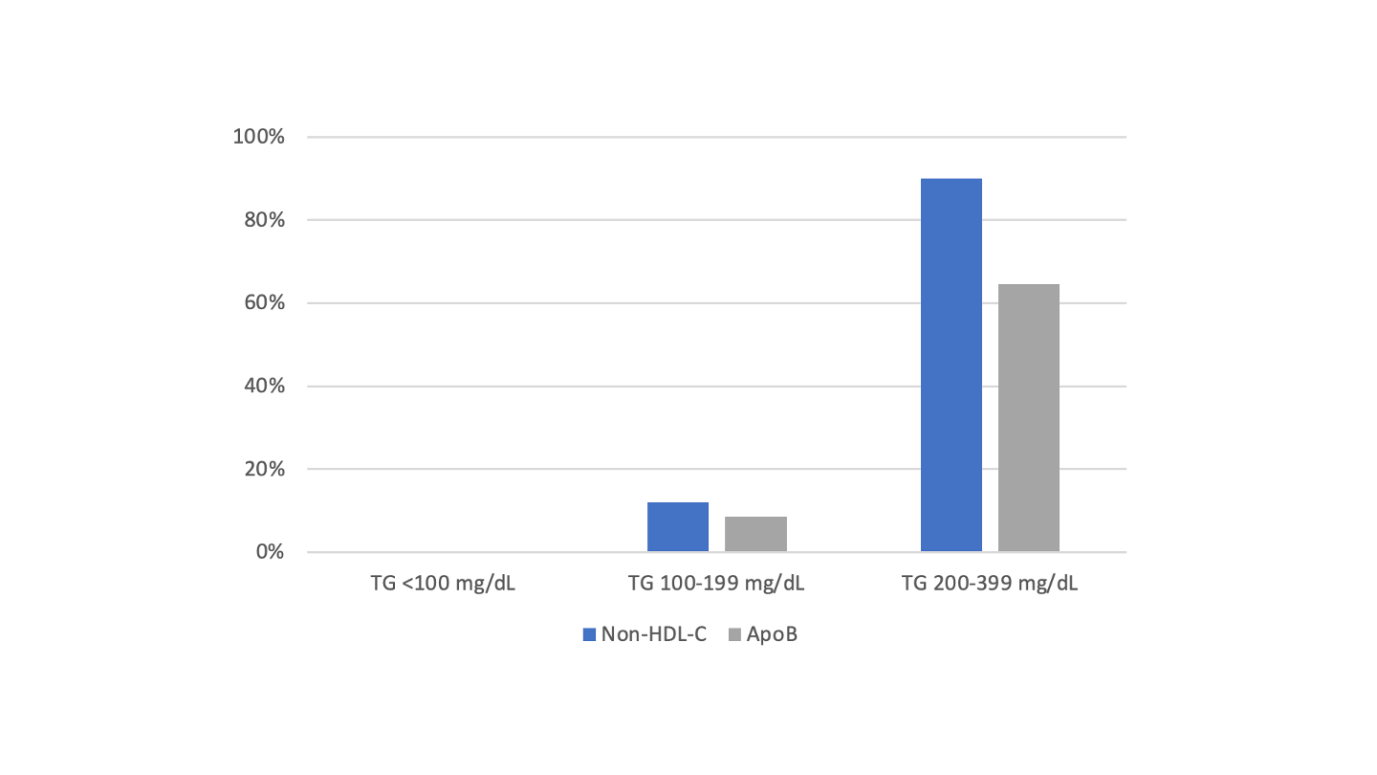
**
